# Synergistic association of insomnia and anxiety with cognitive impairment and dementia in older adults

**DOI:** 10.1002/alz.71853

**Published:** 2026-09-23

**Authors:** Yan Chen, Qianxi Wang, Ling Li, Shixing Qian, Li Bai, Tih Shih Lee, Shaowei Zhang, Yaping Li, Qianqian Guo, Yang Yang, Feng Yan, Lei Feng, Xia Li

**Affiliations:** ^1^ Department of Geriatric Psychiatry Shanghai Mental Health Center, Shanghai Jiao Tong University School of Medicine Shanghai China; ^2^ Alzheimer's Disease and Related Disorders Center Shanghai Jiao Tong University Shanghai China; ^3^ Department of Healthcare‐associated Infection Management Shanghai Mental Health Center, Shanghai Jiao Tong University School of Medicine Shanghai China; ^4^ Neuroscience and Behavioural Disorders Programme Duke‐NUS Medical School Singapore Singapore; ^5^ Department of Psychological Medicine Yong Loo Lin School of Medicine, National University of Singapore Singapore Singapore; ^6^ Centre for Healthy Longevity National University Health System Singapore Singapore; ^7^ Healthy Longevity Translational Research Program Yong Loo Lin School of Medicine, National University of Singapore Singapore Singapore

**Keywords:** anxiety, cognitive impairment, comorbidity, dementia, insomnia, plasma biomarkers, p‐tau217, synergistic interaction

## Abstract

**INTRODUCTION:**

We investigated the synergistic effects of comorbid insomnia and anxiety on cognitive impairment and dementia and explored the underlying plasma biomarker mechanisms.

**METHODS:**

We included adults ≥ 60 years from the cross‐sectional cohort named The Shanghai Action to Prevent Dementia in the Elderly (SHAPE) and used data from the longitudinal United Kingdom (UK) Biobank for validation. Regression models and additive interactions were used to evaluate the associations between comorbid insomnia and anxiety and cognitive outcomes. Biomarker mediation effects (tau phosphorylated at threonine 217 [p‐tau217], glial fibrillary acidic protein [GFAP], and neurofilament light chain [NfL]) were assessed in a SHAPE subcohort.

**RESULTS:**

Comorbid insomnia and anxiety were synergistically associated with incident cognitive impairment in the SHAPE cohort. This vulnerability was validated longitudinally in individuals with incident dementia in the UK Biobank cohort. Additionally, p‐tau217 and GFAP mediated this association, predominantly driven by p‐tau217, with GFAP showing a modest but significant independent contribution.

**DISCUSSION:**

Comorbid insomnia and anxiety are synergistically associated with a higher prevalence of cognitive impairment and incidence of dementia. This vulnerability is partially mediated by amyloid pathology, with astrogliosis providing a modest but significant contribution. These findings underscore the need for combined screening and targeted interventions to mitigate dementia risk.

## Highlight

- Neither insomnia nor anxiety alone was linked to cognitive impairment, whereas comorbidity was.
- Their comorbidity showed a synergistic effect and was validated longitudinally for incident dementia.
- The comorbidity and cognition association was mediated mainly by p‐tau217, with additional significant support from GFAP.

## 1 BACKGROUND

Dementia is a growing global health concern, and the number of affected patients is predicted to reach 139 million by 2050.^1^ The Lancet Commission (2024) identified 14 modifiable risk factors that explain an estimated 45% of dementia cases, although more than half of the risk factors for dementia are not yet understood.^2^ In this context, the identification of new and effective targets for early intervention is essential.

Cognitive degeneration has been associated with both insomnia and anxiety, but mixed findings have been reported in epidemiological studies. Some studies describe obvious relationships between insomnia, anxiety, and dementia,^3^, ^4^ whereas other studies report weak or negative relationships,^5^, ^6^, ^7^ perhaps because of confounding. The first limitation is that most studies have examined insomnia and anxiety either independently^8^, ^9^ or as confounding variables, despite the fact that they often occur together and exacerbate one another. This simplification may result in possible synergistic effects being ignored, namely, that joint disturbances of sleep and anxiety stimulate faster cognitive deterioration than either does individually.

However, it remains unclear whether shared insomnia and anxiety represent increased risk factors for cognitive impairment and dementia, and the underlying biological processes are poorly understood. To address this issue, we used two complementary cohorts – the SHAPE cohort, a Chinese community‐based cohort with abundant biomarker information, and the UK Biobank (UKB) cohort, a large‐scale prospective investigative cohort based in the United Kingdom (UK). We established three main objectives. The first objective was to determine whether coexisting insomnia and anxiety are joint risk factors for cognitive impairment (SHAPE cohort), the second objective was to validate this risk longitudinally in a population with incident dementia (UKB cohort), and the third was to investigate whether the effects of these risk factors are mediated by biomarkers associated with neurodegeneration.

## 2 METHODS

### 2.1 Study design and populations

This dual‐cohort study was conducted following the Strengthening the Reporting of Observational Studies in Epidemiology (STROBE) reporting guidelines. The original SHAPE study was approved by the Institutional Review Board (IRB) of the Shanghai Mental Health Center (No. 2024‐54). The National Health Service North West Multi‐Centre Research Ethics Committee approved the UKB study (Application No. 544746). Written informed consent was obtained from all participants in the two cohorts.

In this study, a cross‐sectional comparison was performed based on the baseline measures of the community‐based SHAPE^10^, ^11^ cohort. Primary data were collected from June 2024 to December 2025 across 16 communities in Shanghai, China. An analysis of the final dataset revealed that 65,857 participants met the following inclusion criteria: (1) age at enrollment was 60 years or older and (2) complete information on the important variables was available (Figure S1A). A biomarker subcohort was composed of 3055 patients, with these patients identified through a random sampling strategy.

UKB is a cohort‐based population study. Baseline measurements were obtained across 22 centers in the UK between 2006 and 2010, and longitudinal follow‐up was performed through linked national registries. The total number of included participants was 149,467 after participants younger than 60 years, those with baseline dementia, and those with missing essential variables were eliminated (Figure S1B).

### 2.2 Assessments in SHAPE cohort

Cohort‐specific definitions were used to measure baseline anxiety and insomnia in the two cohorts. In the SHAPE cohort, exposures were measured through validated questionnaires, namely, an eight‐item Athens Insomnia Scale (AIS‐8) score >6 or the use of sleep medication and a seven‐item Generalized Anxiety Disorder scale (GAD‐7) score of more than 4 (described in the Supplementary Methods). The participants were ultimately divided into 4 baseline phenotypes: no conditions, isolated insomnia, isolated anxiety and comorbid insomnia and anxiety. To further explore the impact of symptom severity, we also performed a semiquantitative analysis. Specifically, the AIS‐8 scores were stratified into three tiers: no insomnia (<4 points), suspected insomnia (4 to 6 points), and clinically significant insomnia (>6 points). Additionally, the GAD‐7 scores were divided into three categories: no anxiety (0 to 4 points), mild anxiety (5 to 9 points), and moderate‐to‐severe anxiety (≥10 points). This cross‐stratification allowed us to further categorize participants into nine subgroups.

**RESEARCH IN CONTEXT**

1. **Systematic review**: We searched PubMed and Google Scholar for studies on insomnia, anxiety, cognitive outcomes, and blood‐based neurodegeneration biomarkers. Prior studies largely assessed insomnia and anxiety separately, with inconsistent findings. Evidence on their joint effects, particularly in relation to p‐tau217 and other plasma biomarkers, remains limited.
2. **Interpretation**: Using a community‐based cross‐sectional cohort and an independent prospective cohort, this study shows that comorbid insomnia and anxiety is associated with cognitive impairment and, on longitudinal validation, with incident dementia, with evidence of synergistic interaction in both settings. Biomarker analyses further suggest that this association is linked primarily to amyloid pathology, while GFAP showed a consistent and statistically significant, though smaller, contribution.
3. **Future directions**: Future research should investigate whether integrated clinical management of this specific comorbidity can mitigate biomarker progression and reduce dementia incidence.

In the analysis of the SHAPE cohort, cognitive impairment was the key outcome. The clinical presentations were used by geriatric psychiatrists and general practitioners to obtain their diagnoses. The 11‐point screening module of the Th oven Cognitive Self‐Assessment (TCSA), a validated electronic cognitive assessment instrument that we previously established,^12^ was used to assess cognition. The overall structure of the TCSA is described in the Supplementary Methods.

### 2.3 Assessments in UKB cohort

Since chronic, clinically significant disease states, as opposed to transient symptoms, should be determined, anxiety in the UKB cohort was characterized using the International Classification of Diseases, Tenth Revision (ICD‐10) code F41 (other anxiety disorders). The diagnosis of insomnia was determined mainly through ICD‐10, using diagnoses of non‐organic insomnia (F51.0) and insomnia (G47.0) associated with the initiation and maintenance of sleep. To further describe chronic sleep disturbance, these diagnostic codes were expanded by adding a self‐reported phenotype of usually experiencing sleeplessness (the detailed data are presented in Table S1). The participants were ultimately divided into four baseline phenotypes: no conditions, isolated insomnia, isolated anxiety, and comorbid insomnia and anxiety.

In the UKB cohort, the incidence of all‐cause dementia (F00‐F03 in Table S1) was the major outcome. Follow‐up was performed until the incidence of dementia, death, or censoring, whichever occurred first.

### 2.4 Covariates

The covariates analyzed in both cohorts included age, sex, education level, smoking status, alcohol use status, and history of hypertension, diabetes, coronary heart disease, and stroke. Baseline depression was determined directly through cohort‐specific criteria: a 15‐item Geriatric Depression Scale score of 5 or higher in the SHAPE cohort and an ICD‐10 diagnosis (codes F32–F33) in the UKB cohort. The models for the UKB cohort and SHAPE biomarker subcohort were also adjusted for the *APOE* ε4 carrier status of the participants. The different measurement criteria and variable definitions used for the two cohorts are presented in the Supplementary Methods (SHAPE cohort) and Table S1 (UKB cohort).

### 2.5 Biomarkers and *APOE* genotyping

All plasma concentrations of tau phosphorylated at threonine 217 (p‐tau217), glial fibrillary acidic protein (GFAP), and neurofilament light chain (NfL) were measured via the AST‐Sc‐Lite platform (AstraBio, Suzhou, China), which is a single‐molecule imaging and detection (SMID) technology.^13^ Detailed protocols, assay comparisons between the AST‐Sc‐Lite and Quanterix platforms, and an evaluation of clinical diagnostic performance are provided in the Supplementary Methods. *APOE* ε4 carrier status was identified by *APOE* genotyping based on quantitative polymerase chain reaction (qPCR) of single‐nucleotide polymorphisms (SNPs) of 429,348 and 7412. The detailed protocols are described in the Supplementary Methods.

### 2.6 Statistical analysis

The baseline characteristics are presented as means with standard deviations (SDs) or frequencies with percentages, as appropriate. Multivariate logistic regression was used to estimate the odds ratios (ORs) of cognitive impairment in the SHAPE cohort. In the UKB cohort, hazard ratios (HRs) of incident dementia were calculated by using Cox proportional hazards regression models. Models were also optimized on the basis of sociodemographic, lifestyle, cardiometabolic, depression, and *APOE* ε4 data.

Interaction on the additive scale was evaluated using the relative excess risk due to interaction (RERI) and the attributable proportion (AP). Because the synergy index (SI) can be validly interpreted only when both exposures act as independent risk factors for the outcome,^14^ and single exposures did not show an independent risk‐increasing effect in the present study, the SI was reported as a sensitivity analysis in the supplementary table. Additionally, parallel multiple mediation models with a multicategory exposure and a binary outcome were estimated using a two‐step regression framework in R using the boot package. Path a, which links the exposure groups to each log‐transformed continuous mediator (log‐transformed p‐tau217, GFAP, and NfL), was modeled via ordinary least squares (OLS) linear regression to obtain unstandardized coefficients. Paths b(mediator–outcome) and $c^{\prime }$(direct exposure–outcome) were modeled simultaneously via logistic regression to obtain coefficients on the log‐odds scale.^15^ Specific indirect effects were quantified as the product of the a and b path coefficients (a×b, log‐odds scale), and the total indirect effect was calculated as the sum of these individual products. Statistical significance was determined using non‐parametric bias‐corrected and accelerated (BCa) bootstrap 95% confidence intervals derived from 5000 resamples.^16^ In the UKB cohort, the population attributable fraction (PAF) was estimated for incident dementia.

We conducted sensitivity analyses, including the sequential removal of participants with previous stroke history, a low education level, and the self‐reported use of sleep medications (SHAPE cohort), as well as the elimination of participants with baseline depression or incident dementia within the first 5 years of follow‐up (UKB cohort).

A two‐sided *p* value of less than 0.05 was considered to indicate statistical significance. Analyses were performed with R, version 4.5.0.

## 3 RESULTS

### 3.1 Attributes of study population

The participants included 65,857 individuals from the SHAPE cohort (mean [SD] age, 72.6 [8.1] years; 27,105 women [41.2%]) and 149,467 individuals from the UKB cohort (mean [SD] age, 63.9 [2.8] years; 72,227 women [48.3%]). Baseline comorbid insomnia and anxiety were reported in 1592 people (2.4%) in the SHAPE cohort and 2785 people (1.9%) in the UKB cohort (Table 1). In both cohorts, compared with people without comorbid insomnia and anxiety, people with comorbid insomnia and anxiety were less educated, smoked more, and were more prone to cardiometabolic diseases. In the SHAPE cohort, comorbid insomnia and anxiety were associated with worse cognitive scores, more frequent cognitive impairment (67.3% vs 29.4%), and greater p‐tau217 and GFAP concentrations in the biomarker subcohort (Table S2). In both cohorts, neither isolated anxiety nor isolated insomnia was significantly related to poor cognitive outcomes.

**TABLE 1:** Baseline characteristics of study populations across dual cohort.

	SHAPE						UK Biobank					
Characteristics	No condition	Isolated insomnia	Isolated anxiety	Comorbid	Total population	*P* value	No condition	Isolated insomnia	Isolated anxiety	Comorbid	Total population	*P* value
	*N *= 42,915	*N *= 20,904	*N *= 446	*N *= 1592	*N *= 65,857		*N *= 103,924	*N *= 41,931	*N *= 827	*N *= 2785	*N *= 149,467	
Age, mean (SD)	71.8 (7.8)	74.0 (8.3)	73.3 (8.2)	78.4 (9.5)	72.6 (8.1)	<0.001	63.9 (2.8)	64.0 (2.8)	63.8 (2.8)	64.1 (2.9)	63.9 (2.8)	<0.001
Female, No. (%)	18,576 (43.3)	7730 (37.0)	201 (45.1)	598 (37.6)	27,105 (41.2)	<0.001	53,090 (51.1)	17,946 (42.8)	306 (37.0)	885 (31.8)	72,227 (48.3)	<0.001
Education Level, No. (%)						<0.001						<0.001
Low	16,093 (37.5)	9954 (47.6)	178 (39.9%)	728 (45.7)	26,953 (40.9)		23,134 (22.3)	10,488 (25.0)	220 (26.6)	761 (27.3)	34,603 (23.2)	
Intermediate	24,930 (58.1)	9885 (47.3)	243 (54.5)	778 (48.9)	35,836 (54.4)		42,782 (41.2)	17,378 (41.4)	347 (42.0)	1151 (41.3)	61,658 (41.3)	
High	1892 (4.4)	1065 (5.1)	25 (5.6)	86 (5.4)	3068 (4.7)		38,008 (36.6)	14,065 (33.5)	260 (31.4)	873 (31.3)	53,206 (35.6)	
Smoking status, No. (%)						<0.001						<0.001
Never	NA	NA	NA	NA	NA		54,953 (52.9)	20,951 (50.0)	414 (50.1)	1312 (47.1)	77,630 (51.9)	
Previous	NA	NA	NA	NA	NA		41,541 (40.0)	18,208 (43.4)	334 (40.4)	1234 (44.3)	61,317 (41.0)	
Current	4849 (11.3)	2373 (11.4)	72 (16.1)	231 (14.5)	7525 (11.4)		7430 (7.1)	2772 (6.6)	79 (9.6)	239 (8.6)	10,520 (7.0)	
Alcohol consumption, No. (%)						<0.001						<0.001
Never	NA	NA	NA	NA	NA		4135 (4.0)	1793 (4.3)	45 (5.4)	161 (5.8)	6134 (4.1)	
Previous	NA	NA	NA	NA	NA		2881 (2.8)	1537 (3.7)	57 (6.9)	177 (6.4)	4652 (3.1)	
Current	3749 (8.7)	2055 (9.8)	44 (9.9)	195 (12.2)	6043 (9.2)		96,908 (93.2)	38,601 (92.1)	725 (87.7)	2447 (87.9)	138,681 (92.8)	
Coronary heart disease, No. (%)	2372 (5.5)	2323 (11.1)	46 (10.3)	259 (16.3)	5000 (7.6)	<0.001	2477 (2.4)	1069 (2.5)	23 (2.8)	87 (3.1)	3656 (2.4)	0.05
Stroke history, No. (%)	1286 (3.0)	1419 (6.8)	24 (5.4)	164 (10.3)	2893 (4.4)	<0.001	732 (0.7)	325 (0.8)	7 (0.8)	20 (0.7)	1084 (0.7)	0.69
Hypertension, No. (%)	12,601 (29.4)	9697 (46.4)	166 (37.2)	859 (54.0)	23,323 (35.4)	<0.001	30,509 (29.4)	13,431 (32.0)	281 (34.0)	949 (34.1)	45,170 (30.2)	<0.001
Diabetes, No. (%)	4221 (9.8)	3483 (16.7)	56 (12.6)	419 (26.3)	8179 (12.4)	<0.001	6190 (6.0)	3129 (7.5)	51 (6.2)	222 (8.0)	9592 (6.4)	<0.001
Depression, No. (%)	2841 (6.6)	4650 (22.2)	188 (42.2)	1201 (75.4)	8880 (13.5)	<0.001	3668 (3.5)	2317 (5.5)	358 (43.3)	1209 (43.4)	7552 (5.1)	<0.001
*APOE* ε4 carrier, No. (%)	NA	NA	NA	NA	NA	NA	29,350 (28.2)	11,507 (27.4)	243 (29.4)	789 (28.3)	41,889 (28.0)	0.04
TCSA score, mean (SD)	8.36 (2.67)	7.84 (2.75)	8.42 (2.65)	6.20 (3.19)	8.14 (2.74)	<0.001	NA	NA	NA	NA	NA	NA
Cognitive impairment, No. (%)	12,604 (29.4)	7568 (36.2)	156 (35.0)	1071 (67.3)	21,399 (32.5)	<0.001	NA	NA	NA	NA	NA	NA
Dementia, No. (%)	NA	NA	NA	NA	NA	NA	2623 (2.5)	1067 (2.5)	43 (5.2)	210 (7.5)	3943 (2.6)	<0.001

*Note*: The data were expressed in the form of mean (SD), numbers, and percentages. The Kruskal‐Wallis test was used to carry out group comparisons of continuous variables. The comparison of categorical variables was done through the chi‐squared test (or Fisher's exact test where necessary). Strictly defined exposure phenotypes were used depending on clinical status at baseline. To strictly avoid reverse causation and immortal time bias, a time‐dependent analysis of them was conducted, letting those participants who developed such symptoms during the period they were followed up in a baseline follow‐up be included in the time‐dependent analysis and not assigned retrospectively to the baseline follow‐up. Some were cohort variables as denoted by NA.

Abbreviations: APOE, apolipoprotein E; CHD, coronary heart disease; SD, standard deviation; TCSA, Thoven cognitive self‐assessment.

### 3.2 Synergistic association of cognitive impairment in SHAPE cohort

**FIGURE 1:**
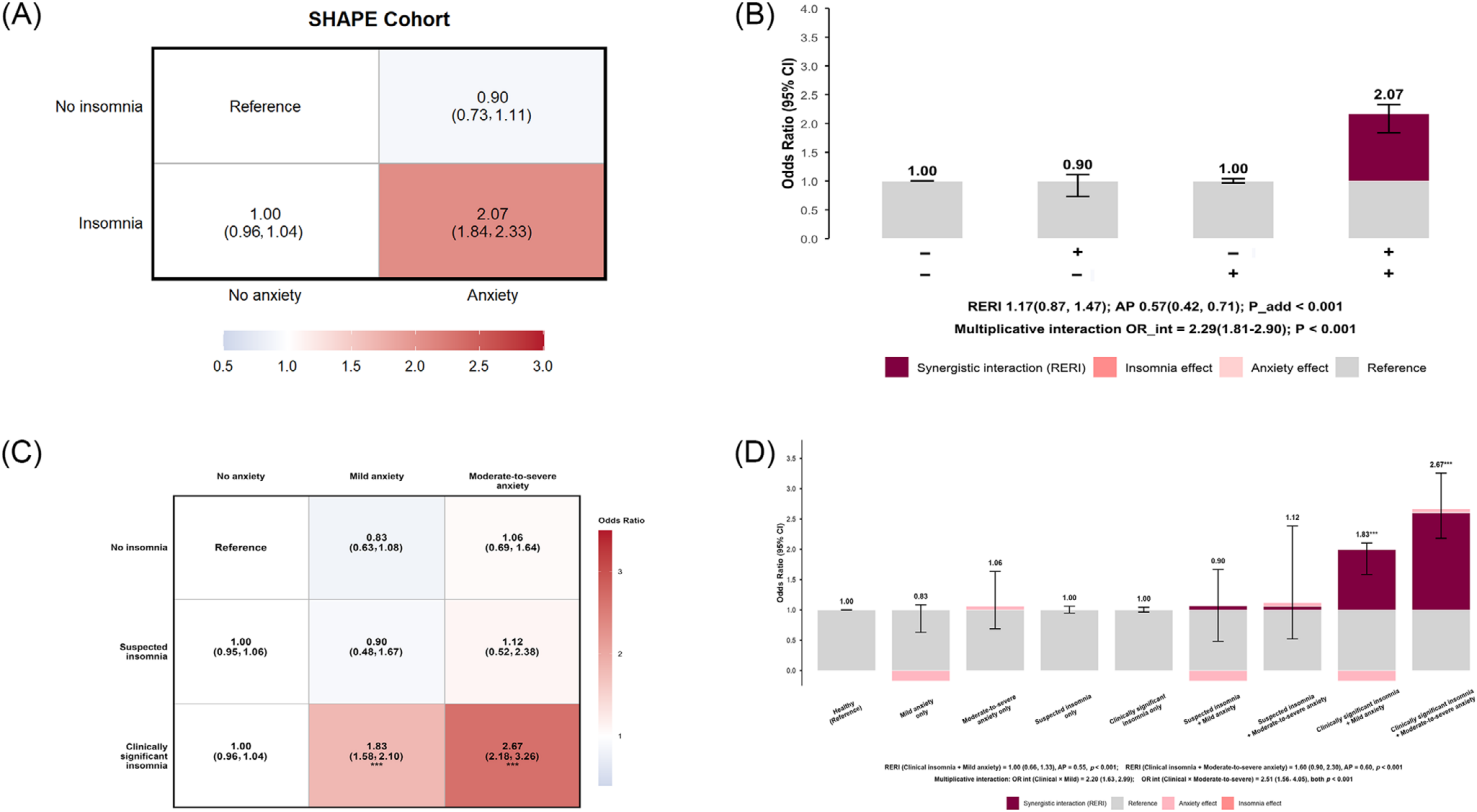
Synergistic association between comorbid insomnia and anxiety and cognitive impairment in the SHAPE cohort. (A) Heatmap of cross‐sectional relationships of insomnia and anxiety phenotypes with cognitive impairment in SHAPE cohort. The values are the ORs and 95% CIs determined on the basis of multivariate logistic regression models. The color gradient shows the level of risk, with blue representing the lowest risk and deep red representing the highest risk compared with the reference group (no condition). (B) Stacked bar chart of cross‐sectional OR for cognitive impairment in SHAPE cohort. In the comorbid group (rightmost bar), the overall height is the cumulative risk estimate and is broken down into the baseline reference risk (gray), the independent effect of anxiety (light pink), the independent effect of insomnia (salmon), and an excess risk that is attributable solely to the synergistic interaction of the two conditions on the additive scale (dark red). The error bars represent the 95% CIs of the total risk estimate of each group. (C) Extended heatmap showing cross‐sectional relationships of graded insomnia severity (no insomnia, suspected insomnia, and clinically significant insomnia) and graded anxiety severity (no anxiety, mild anxiety, and moderate to severe anxiety) with cognitive impairment. (D) Stacked bar chart corresponding to panel (C) depicting the decomposed risk components across the full spectrum of insomnia and anxiety severity combinations. The additive and multiplicative interactions are indicated by the statistical indices presented below each panel. AP, attributable proportion; CI, confidence interval; OR, odds ratio; RERI, relative excess risk due to interactions.

In the cross‐sectional SHAPE study, comorbid insomnia and anxiety were the only comorbidities associated with incident cognitive impairment (OR = 2.07, 95% CI: 1.84 to 2.33; Figure 1A). The additive interaction (RERI = 1.17, 95% CI: 0.87 to 1.47, *p* < 0.001) and multiplicative interaction (OR = 2.29, 95% CI: 1.81 to 2.90, *p* < 0.001) between comorbid insomnia and anxiety and cognitive impairment were significant (Figure 1B). The AP was 0.57, indicating that this interaction caused 57% of the risk of cognitive impairment in individuals with comorbid anxiety and insomnia. All interaction measures, including the SI, are reported in Table S3. These results were consistent with those of the biomarker subcohort (Figure S2).

Further semiquantitative analyses were performed to explore the impact of symptom severity, and the results (Figure 1C,D) yielded more detailed insights. No significant associations with incident cognitive impairment were observed for any of the subclinical combinations. Specifically, suspected insomnia with mild anxiety had an OR of 0.90 (95% CI: 0.48 to 1.67), whereas suspected insomnia co‐occurring with moderate to severe anxiety had an OR of 1.12 (95% CI: 0.52 to 2.38). In contrast, neither mild anxiety alone (OR = 0.83, 95% CI: 0.63 to 1.08) nor moderate to severe anxiety alone (OR = 1.06, 95% CI: 0.69 to 1.64) deviated from the reference risk. An elevated risk of cognitive impairment was detected only among participants with both clinically significant insomnia and anxiety, with the strength of this association increasing in a stepwise manner with increasing anxiety severity. Specifically, the combination of clinically significant insomnia with mild anxiety had an OR of 1.83 (95% CI: 1.58 to 2.10, *p* < 0.001), whereas the combination with moderate to severe anxiety had an OR of 2.67 (95% CI: 2.18 to 3.26, *p* < 0.001) (Figure 1C,D). These combinations were also associated with significant additive interactions (RERI = 1.00, AP = 0.55, *p* < 0.001 for clinically significant insomnia with mild anxiety; RERI = 1.60, AP = 0.60, *p* < 0.001 for clinically significant insomnia with moderate to severe anxiety) and large multiplicative interactions (OR = 2.20, 95% CI: 1.63 to 2.99 and OR = 2.51, 95% CI: 1.56 to 4.05, respectively; both *p* < 0.001), underscoring that the synergistic effect of comorbid insomnia and anxiety on the association of cognitive impairment remains robust across different severity levels and depends on the cumulative clinical‐level symptom burden.

### 3.3 Longitudinal confirmation of incident dementia in UKB cohort

**FIGURE 2:**
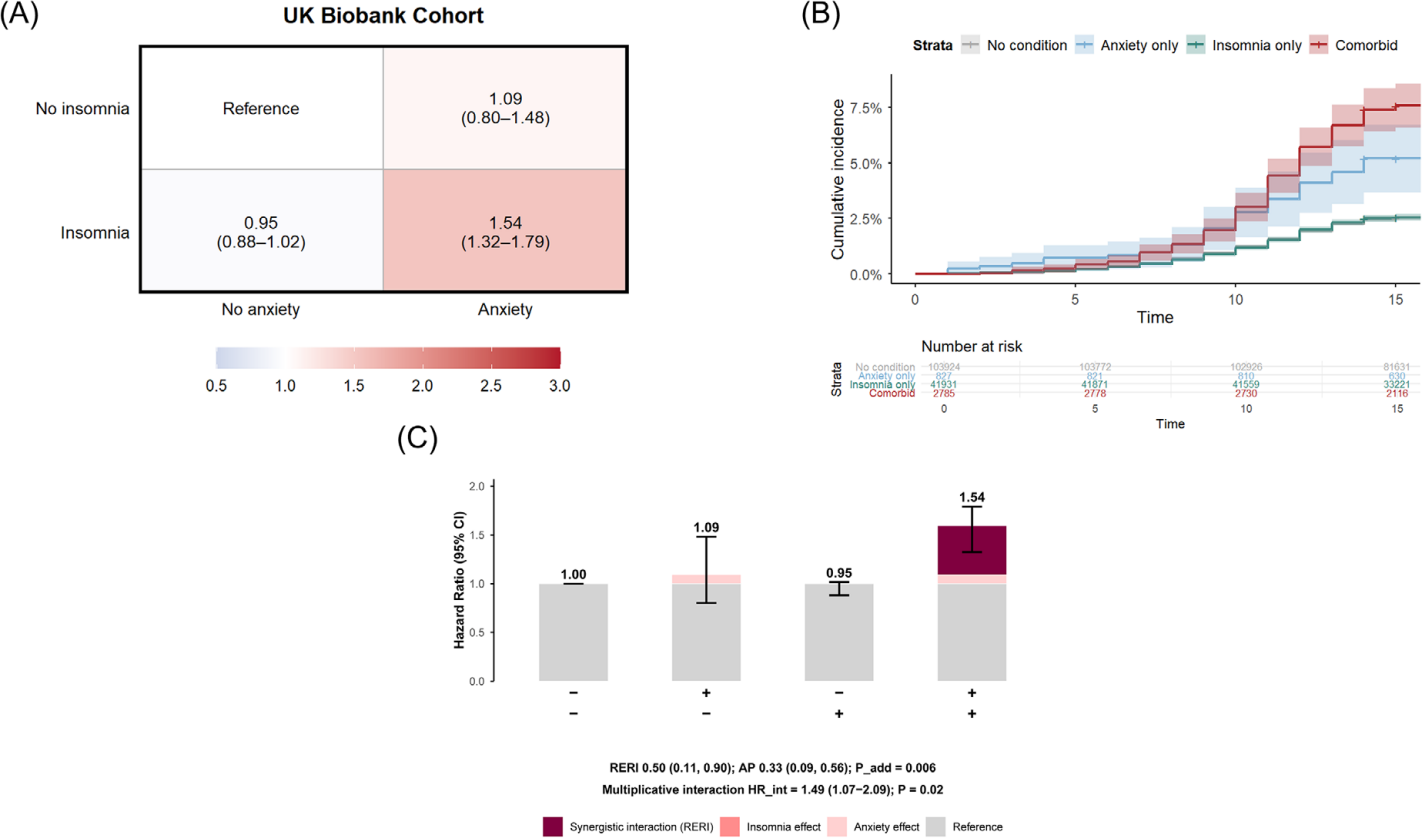
Synergistic association between comorbid insomnia and anxiety and incident dementia in UK Biobank cohort. (A) Heatmap of longitudinal risk of incident dementia in UK Biobank cohort. The values are the HRs and 95% CIs, which were derived through multivariate Cox proportional hazard models. The color gradient shows the level of risk, with blue indicating the lowest risk and deep red the highest risk compared with the reference group (no condition). (B) Cumulative incidence curves of dementia in UK Biobank cohort over a 15‐year follow‐up period by clinical phenotype at baseline. The shaded areas represent the 95% CIs. The population at risk in the 5‐year intervals is indicated below the plot. All the models were modified to include sociodemographic factors, lifestyle factors, baseline clinical comorbidities, and *APOE* ε4 carrier status. (C) Stacked bar chart of total risk (HR) of incident dementia in UK Biobank cohort. The bar number indicates the clinical phenotype, which depends on the presence or absence of anxiety and insomnia. In the comorbid group (rightmost bar), the overall height is the cumulative risk estimate and is broken down into the baseline reference risk (gray), the independent effect of anxiety (light pink), the independent effect of insomnia (salmon), and an excess risk that is attributable solely to the synergistic interaction of the two conditions on the additive scale (dark red). The error bars represent the 95% CIs of the total risk estimate of each group. The additive and multiplicative interactions are indicated by the statistical indices provided below each panel. AP, attributable proportion; CI, confidence interval; HR, hazard ratio; OR, odds ratio; RERI, relative excess risk due to interactions.

**FIGURE 3:**
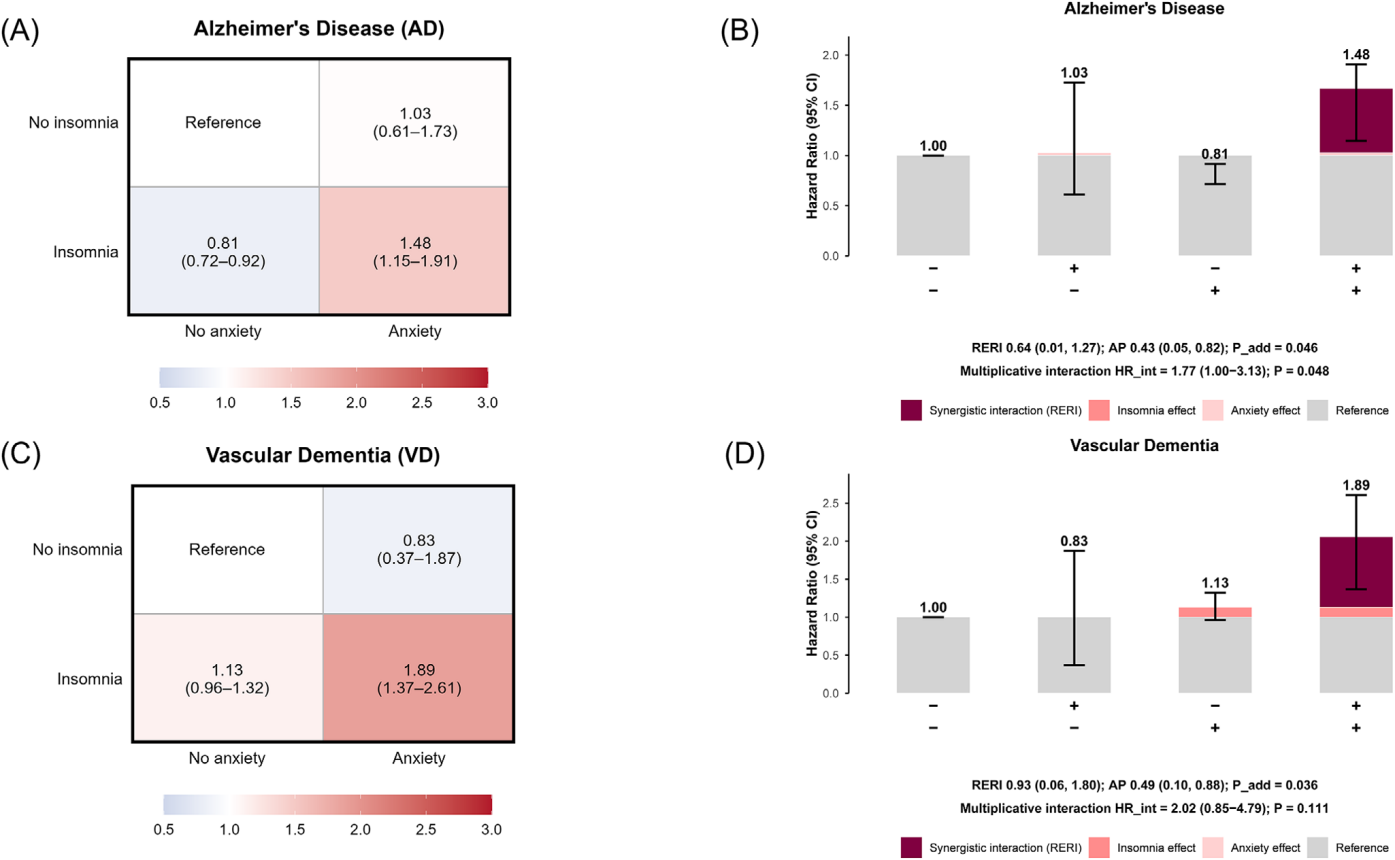
Synergistic association between comorbid insomnia and anxiety and incident dementia subtypes in UK Biobank cohort. (A) Heatmap of longitudinal risk of incident AD and (C) VD in UK Biobank cohort. The values are given as HRs and 95% CIs, which were derived through multivariate Cox proportional hazards models. The color gradient shows the level of risk, with blue representing the lowest risk and deep red the greatest risk compared with the reference group (no condition). (B and D) Stacked bar charts of total risk (HR) of incident AD (B) and VD (D) in UK Biobank cohort. The bar number indicates the clinical phenotype, which depends on the presence or absence of anxiety and insomnia. In the comorbid group (rightmost bar), the overall height is the cumulative risk estimate and is broken down into the baseline reference risk (gray), the independent effect of anxiety (light pink), the independent effect of insomnia (salmon), and an excess risk that is attributable solely to the synergistic interaction of the two conditions on the additive scale (dark red). The error bars represent the 95% CIs of the total risk estimate of each group. The additive and multiplicative interactions are indicated by the statistical indices provided below each panel. AD, Alzheimer's disease; AP, attributable proportion; CI, confidence interval; HR, hazard ratio; RERI, relative excess risk due to interactions; VD, vascular dementia.

To address the cross‐sectional limitations of the discovery cohort and assess the long‐term prognostic value, we longitudinally evaluated this vulnerability in the UKB cohort. The comorbid phenotype was associated with incident dementia (HR = 1.54, 95% CI: 1.32 to 1.79), but individual anxiety and insomnia symptoms were not (Figure 2A). The cumulative incidence of dementia over a 15‐year follow‐up period was significantly greater in the comorbid group than in the isolated‐condition and reference groups (Figure 2B). Moreover, this comorbidity had a strong additive effect on the incidence of dementia (RERI = 0.50, 95% CI: 0.11 to 0.90, *p* = 0.006) in the UKB cohort (AP = 0.33) (Figure 2C), as well as a large multiplicative effect (HR = 1.49, 95% CI: 1.07 to 2.09, *p* = 0.02). This synergistic pattern was further pronounced when patients were stratified by dementia subtype. Specifically, among patients with Alzheimer's disease (AD), the HR of the comorbid group was 1.48 (95% CI: 1.15 to 1.91), with a significant additive effect (RERI = 0.64, AP = 0.43, *p *= 0.046) and multiplicative interaction effect (HR = 1.77, *p *= 0.048) (Figure 3A,B). Among patients with vascular dementia (VD), the HR of the comorbid group reached 1.89 (95% CI: 1.37 to 2.61), with a substantial additive interaction effect (RERI = 0.93, AP = 0.49, *p *= 0.036); however, the multiplicative interaction effect was not statistically significant in this group (HR = 2.02, *p *= 0.111) (Figure 3C,D).

### 3.4 Biomarker associations and mediation analysis

In the SHAPE biomarker subcohort, the comorbid phenotype was associated with elevated p‐tau217 (unstandardized *b* = 0.42, 95% CI: 0.31 to 0.52, *p* < 0.001) and GFAP (unstandardized *b* = 0.24, 95% CI: 0.10 to 0.39, *p* = 0.001) concentrations, whereas no significant association with the NfL concentration was observed (unstandardized *b* = −0.14, 95% CI: −0.43 to 0.16, *p* = 0.356). Neither isolated anxiety nor isolated insomnia was significantly associated with the levels of any biomarker (all *p* > 0.05) (Table S4).

**FIGURE 4:**
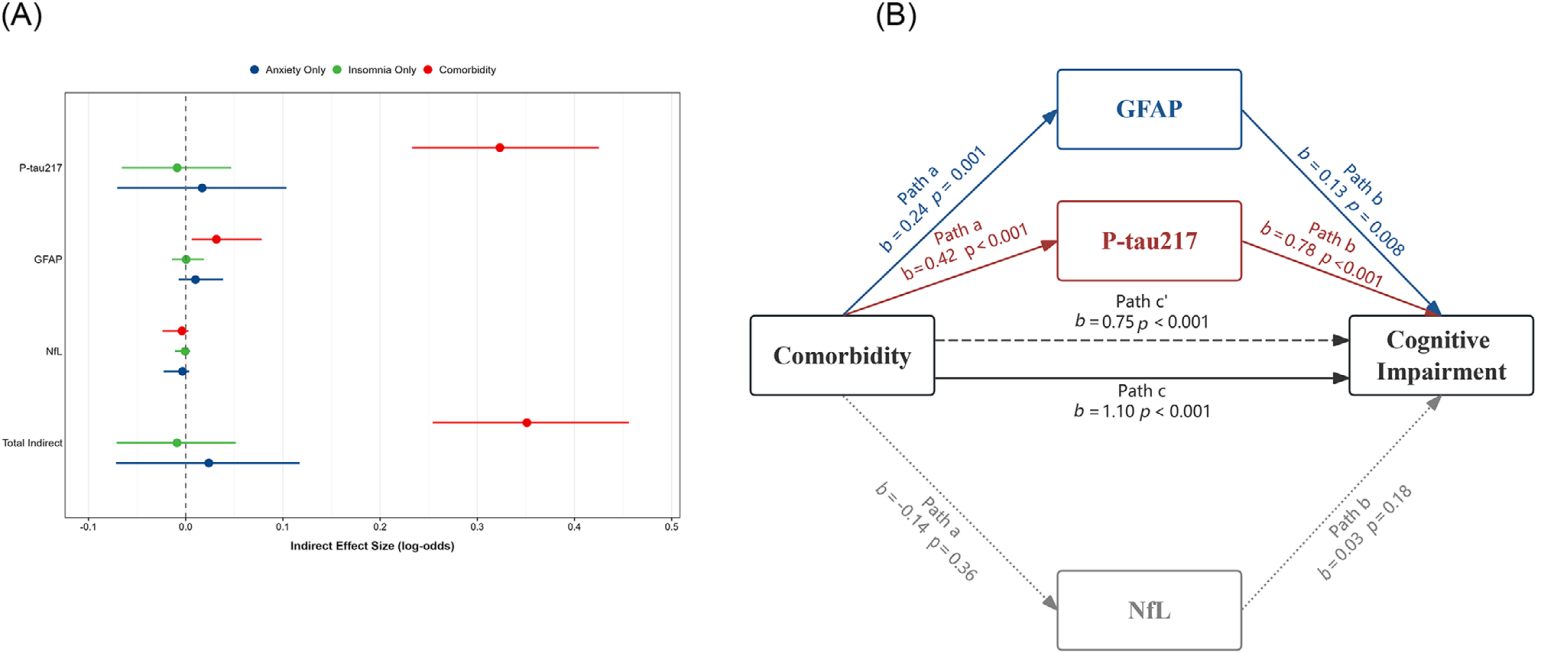
Parallel mediation of plasma biomarkers in relationship between insomnia–anxiety phenotypes and cognitive impairment in SHAPE subcohort. (A) Forest plot of specific indirect effects and total indirect effects for each mediator (p‐tau217, GFAP, and NfL) across the three groups. The error bars represent the 95% confidence intervals based on 5000 BCa bootstrap resamples. (B) Path diagram for comorbidity group showing unstandardized path coefficients (b). Path a represents the linear regression coefficients from the independent variable to the mediators; paths b, c', and c represent log‐odds determined via logistic regression. The solid black arrow (path C) represents the total effect, and the dashed black arrow (path C') represents the direct effect, that is, the direct effect without the mediators. The sociodemographic factors, lifestyle factors, baseline clinical comorbidities, and *APOE* ε4 carrier status were adjusted in all the mediation models. GFAP, glial fibrillary acidic protein; NfL, neurofilament light; p‐tau217, tau phosphorylated at threonine 217.

In the parallel mediation model for the comorbid group, p‐tau217 emerged as the principal mediator, with a *b* value of 0.32 (log‐odds, 95% CI: 0.233 to 0.425). Additionally, GFAP demonstrated a relatively small but significant independent contribution (*b* = 0.03, log‐odds, 95% CI: = 0.006 to 0.078). In contrast, the indirect effect through the NfL pathway was not significant (*b* = −0.004, log‐odds, 95% CI: −0.024 to 0.003) (Figure 4A). The combined biomarker panel yielded a significant total indirect effect of *b* = 0.35 (log‐odds, 95% CI: 0.254 to 0.456), mediating the total effect of the comorbid phenotype on cognitive impairment (Path c: *b* = 1.10, log‐odds, 95% CI: 0.772 to 1.398). Notably, no significant biomarker‐mediated pathways were observed for either anxiety alone or insomnia alone (Figure 4B). The detailed parameters are provided in Table S4.

### 3.5 Population attributable fraction

The prevalence of comorbid insomnia and anxiety among patients with incident dementia in the UKB cohort was 5.33%. The estimated PAF of the comorbid phenotype, determined on the basis of the fully adjusted Cox models, was 1.87% (95% CI: 1.31 to 2.36%).

### 3.6 Sensitivity analyses

The results of the sensitivity analyses were consistent with the above findings (Tables S5–S9). The synergetic effect of comorbid anxiety and insomnia was maintained in the SHAPE cohort following the sequential elimination of participants who had a previous stroke, participants who had a low level of education, and participants who were taking sleep medication. Furthermore, the association remained statistically significant in the UKB cohort when the participants who had baseline depression or who displayed incident dementia during the first 5 years of follow‐up were excluded from the analysis.

## 4 DISCUSSION

In this dual‐cohort study, we first identified a synergistic association between comorbid insomnia and anxiety and incident cognitive impairment and then validated this comorbid state as a longitudinal predictor of incident dementia, including both AD and VD subtypes. Importantly, we determined that underlying brain amyloid pathology and astrogliosis partially mediated this synergistic vulnerability.

Studies in which anxiety and insomnia have been examined individually, rather than in combination, in relation to dementia risk have yielded inconsistent results. Several studies reported that both anxiety and insomnia were risk factors for dementia^5^, ^17^; however, other studies that controlled for additional covariates or employing Mendelian randomization approaches reported that neither exposure factor independently increased the risk of dementia.^6^, ^18^, ^19^, ^20^ We aimed to address this inconsistency by examining two cohorts, revealing that neither anxiety alone nor insomnia alone appeared to significantly increase the risk of cognitive decline; however, comorbid anxiety and insomnia did increase this risk. This conclusion was supported through two sets of observations made in different contexts, namely, subclinical symptoms evaluated cross‐sectionally in the SHAPE cohort and chronic clinical diagnoses documented longitudinally in the UKB cohort. Our supplementary semiquantitative severity stratification analyses further support this dose‒response pattern. Specifically, the synergistic association between comorbid anxiety and insomnia and the increased association of cognitive impairment was observed only when clinically significant insomnia co‐occurred with anxiety, and the risk of cognitive decline increased in a stepwise manner with increasing anxiety severity, with the significant additive and multiplicative interaction effects between the two symptoms remaining stable in the stratified framework. No excess risk of cognitive impairment was detected for patients with both subclinical (suspected) insomnia and anxiety of any severity level, indicating that the synergistic effect depends on the cumulative symptom burden. This severity‐dependent pattern also partially explains why insomnia and anxiety alone are insufficient to constitute risk factors for dementia. These findings indicate that the brain can effectively compensate for sleep disturbances and anxiety individually. For example, intact stress response systems could be important for sleep loss resilience,^21^ and a preserved sleep architecture could be useful for preventing the adverse consequences of psychological distress.^22^

This compensatory potential, however, fails when insomnia and anxiety co‐occur according to our interaction analyses. The combination of the two conditions likely results in a vicious cycle: Hyperarousal and intermittent activation of the hypothalamic–pituitary–adrenal (HPA) axis, caused by anxiety, fragments sleep,^23^ whereas poor sleep results in the inability of the prefrontal cortex to control emotions.^24^ This vicious cycle negatively affects physiological resources and leads to the development of a neurotoxic environment, enhancing the progression toward dementia.

The results of the biomarker and mediation analyses elucidate the complex biological cascade linking comorbid insomnia and anxiety to cognitive decline. The observation that both plasma p‐tau217 and GFAP significantly mediate this association suggests a convergent “multi‐hit” pathological model in which psychological stress and sleep disturbance synergistically drive core hallmarks of AD. The significant mediation by plasma p‐tau217 underscores the central role of cerebral amyloid beta (Aβ) pathology in this process. Our findings suggest that the comorbid state accelerates Aβ accumulation through two distinct but complementary mechanisms: Chronic anxiety results in the activation of the HPA axis, increasing the levels of glucocorticoids that upregulate beta‐secretase activity and promote Aβ production.^25^ Concurrently, insomnia – particularly sleep fragmentation – impair the glymphatic system, which underlies the clearance of waste by the brain, thereby reducing the nocturnal efflux of soluble Aβ peptides.^26^ The combined effect of increased production and decreased clearance of Aβ likely explains the robust increase in p‐tau217 levels observed in the comorbid group. Moreover, the regulation by GFAP highlights the critical contribution of reactive astrogliosis. Specifically, the increase in GFAP levels in our cohort likely represents a specific astrocytic response to the accumulating Aβ and direct synaptic stress induced by sleep deprivation. Emerging evidence suggests that in the presence of Aβ, astrocytes undergo a phenotypic shift, yielding neurotoxic reactive astrocytes, which lose their normal homeostatic functions, such as synaptic support and neuronal metabolic regulation, and may gain neurotoxic properties.^27^ Therefore, the dual mediation effect suggests a pathophysiological sequence in which comorbid insomnia and anxiety initiates a vicious cycle: Stress‐related sleep dysregulation drives Aβ accumulation, which in turn triggers reactive astrogliosis. This reactive state may further exacerbate neuronal dysfunction by impairing synaptic plasticity and neuronal survival, independently of or synergistically with Aβ toxicity. This interplay between amyloidosis and maladaptive astrocytic reactivity provides a compelling biological explanation for the accelerated cognitive decline observed in patients suffering from both insomnia and anxiety.

Nevertheless, these biomarker‐based findings do not necessarily indicate that comorbid insomnia and anxiety affect only AD‐related pathology. Our population‐based analysis of the UKB cohort revealed that comorbid insomnia and anxiety are risk factors for both AD and VD, suggesting that their detrimental effects extend beyond the amyloid‐centric pathway captured by our biomarker analyses. Notably, the synergistic association with VD was at least as pronounced as that with AD, accompanied by robust additive interaction effects. These findings suggest that comorbid insomnia and anxiety may compromise cerebrovascular integrity through pathways operating in parallel to amyloid accumulation pathways. Specifically, chronic activation of the HPA axis and sustained sympathetic arousal increase blood pressure^28^ and impair endothelial function,^29^ whereas sleep deprivation promotes systemic inflammation and blood–brain barrier dysfunction,^30^ collectively accelerating the small‐vessel pathology that underlies VD.^31^ The consistent additive interactions observed across both subtypes imply that this comorbidity drives neurodegeneration through both shared mechanisms, such as neuroinflammation and barrier disruption, and subtype‐specific pathways. Finally, how this comorbid state influences other dementia subtypes remains to be clarified and warrants further investigation in future studies.

These results have important clinical implications. Insomnia and anxiety are often treated synergistically by clinicians to alleviate distress, but the causal relationship between these conditions, which represent unique neurodegenerative risk factors, is often ignored. Our study revealed that the co‐occurrence of insomnia and anxiety is characteristic of a high‐risk subtype of dementia. Thus, the management of a single condition alone might not be sufficient for minimizing long‐term cognitive risk. In older adults with both symptoms, frequent cognitive assessments and combined therapies, such as cognitive behavioral therapy aimed at sleep and emotional control, may not only alleviate psychiatric symptoms but also prevent the development of dementia.

Although the proportion of the population with this comorbidity in the UKB cohort was small, this likely does not reflect the actual societal burdens associated with this comorbidity because the participants in the cohort were volunteers who were healthier than individuals in the general population. Insomnia and anxiety are treatable through behavioral and pharmaceutical methods, in contrast to *APOE* ε4, a genetic risk factor that is immutable. The identification and management of this comorbid phenotype is thus an effective and viable approach that can be scaled to reduce dementia risk at the population level.

A methodological consideration concerns the choice of interaction measures. We based our inference on additive interaction on the RERI and AP. Because the synergy index can be reliably interpreted only when both exposures act as independent risk factors – a condition not encountered in our study, where insomnia and anxiety alone were not significantly associated with risk – we treated the synergy index as a sensitivity analysis rather than a primary measure, in line with established methodological guidance.^14^ In this analysis, the synergy index indeed yielded unstable estimates with uninformative confidence intervals, whereas the RERI and AP were stable and mutually consistent across both cohorts.

This study has several strengths. Most importantly, two separate cohorts were used in the analysis, which allowed us to compare results across populations based on extremely different study designs. The reliability of our conclusions is substantially strengthened by the complementary nature of our exposure definitions. Highly consistent synergistic effects were observed regardless of whether insomnia and anxiety were evaluated as subclinical symptoms via screening scales (SHAPE cohort) or as severe, clinical diagnoses via ICD‐10 codes (UKB cohort), highlighting the robust and universal nature of this comorbidity‐driven cognitive risk. Additionally, the plasma biomarker levels provided a plausible biological model for evaluating the epidemiological observations.

However, this study also has several limitations that should be acknowledged. First, the biomarker levels in the SHAPE cohort were measured at only a single time point, precluding definitive conclusions regarding how p‐tau217 and GFAP evolve over time in relation to comorbid insomnia and anxiety. Second, detailed classifications of the psychotropic medications used by the participants, particularly the specific types of insomnia‐related sedatives (i.e., hypnotics and anxiolytics), and objective sleep measures were not available; consequently, the confounding effects of medication use on the analysis of the anxiety–insomnia–cognition association cannot be fully excluded. We plan to collect detailed medication classifications in our future cohort studies to address this limitation. Third, our investigation of the underlying pathological mechanisms was constrained. Although we leveraged available baseline Olink proteomic and nuclear magnetic resonance (NMR) metabolomic data within a UKB subcohort to probe astrocytic and vascular pathways, these preliminary analyses were heavily underpowered due to the restricted sample sizes of specific subgroups. Consequently, directional trends – such as elevated astrocytic markers and altered lipid‐metabolic profiles in the comorbid group – did not reach statistical significance after multiple‐testing correction. Furthermore, our investigation of mechanisms specific to VD was limited by the absence of highly specific vascular markers (such as homocysteine) in the full cohort. Future research in cohorts with a higher prevalence of vascular cognitive impairment, alongside repeated measures of p‐tau217 closer to clinical onset, is warranted to fully disentangle the comorbid‐specific pathways.

## CONFLICT OF INTEREST STATEMENT

The authors declare that this study was conducted in the absence of any commercial or financial relationships that could be construed as potential conflicts of interest. Author disclosures are available in the supporting information. Author disclosures are available in the Supporting Information.

## CONSENT STATEMENT

All participants in both cohorts provided informed consent.

## Supporting information



Supporting Information

Supporting Information

Supporting Information

Supporting Information
