# Supplementary material for: Synergistic association of insomnia and anxiety with cognitive impairment and dementia in older adults
Source: Alzheimers Dement. 2026 Sep 23;22(9):e71853. doi: 10.1002/alz.71853 (PMC13598542; doi:10.1002/alz.71853)
Supplement: Supplementary file 2 [file ALZ-22-e71853-s001.docx]

**Supplementary Figure 1. Study Flow in the SHAPE and UK Biobank Cohorts**

(A) Flow diagram for the SHAPE cohort (n = 65,857) and biomarker subcohort (n = 3,055) after excluding duplicates, age <60 years, and missing data. (B) Flow diagram for the UK Biobank cohort (n = 149,467) after excluding age <60 years, missing covariates, baseline dementia, and missing APOE genotyping.

**Supplementary Figure 2. Synergistic Interaction of Insomnia and Anxiety on Cognitive Impairment in the SHAPE Cohort**

Figure 2. Synergistic Interaction of Insomnia and Anxiety on Cognitive Impairment in the SHAPE Cohort

(A) Heatmap displaying the cross-sectional association of insomnia and anxiety phenotypes with cognitive impairment in the SHAPE cohort. Values represent odds ratios (ORs) and 95% CIs derived from multivariable logistic regression models. (B) Stacked bar chart decomposing the total risk (odds ratio [OR]) for cross-sectional cognitive impairment into its constituent components. Each bar represents a specific clinical phenotype: neither condition (-, -), isolated insomnia (+, -), isolated anxiety (-, +), and comorbid insomnia and anxiety (+, +). For the comorbid group (rightmost bar), the total height represents the combined risk estimate, which is partitioned into the baseline reference risk (gray), the independent effect of anxiety (light pink), the independent effect of insomnia (salmon), and the excess risk strictly attributable to their synergistic interaction on the additive scale (dark red). Error bars indicate 95% CIs for the total risk estimate of each group. Statistical indices for additive interaction (relative excess risk due to interaction [RERI] and attributable proportion [AP]) and multiplicative interaction are detailed below the panel.

Abbreviations: AP, attributable proportion; OR, odds ratio; RERI, relative excess risk due to interaction.
