# Supplementary material for: Synergistic association of insomnia and anxiety with cognitive impairment and dementia in older adults
Source: Alzheimers Dement. 2026 Sep 23;22(9):e71853. doi: 10.1002/alz.71853 (PMC13598542; doi:10.1002/alz.71853)
Supplement: Supplementary file 3 [file ALZ-22-e71853-s003.docx]

**Table S1 Data Fields for Variables in the UKB Cohort**

| Indicator | Data Field | Description |
| --- | --- | --- |
| Dementia | 41270 | Hospital inpatient diagnoses (ICD-10 codes: F00 [dementia in Alzheimer disease], F01 [vascular dementia], F02 [dementia in other diseases classified elsewhere], and F03 [unspecified dementia]) |
| Anxiety | 41270 | Hospital inpatient diagnoses (ICD-10 codes F41 [other anxiety disorders]) |
| Insomnia | 41270 | Hospital inpatient diagnoses (ICD-10 codes: F51.0 [nonorganic insomnia] and G47.0 [disorders of initiating and maintaining sleep]) |
|  | 1200 | Self-reported response of "Usually" to the touchscreen question: "Do you have trouble falling asleep at night or do you wake up in the middle of the night?" |
| Age | 21022 | Age at recruitment |
| Sex | 31 | Sex |
| Education | 6138 | Self-reported qualifications categorized as low (O levels/GCSEs, CSEs, or equivalent), intermediate (A levels/AS levels, NVQ, HND, HNC, other professional qualifications [eg, nursing, teaching], or equivalent), and high (college or university degree) |
| Smoking Status | 20116 | Self-reported smoking status (categorized as never, previous, and current) |
| Alcohol Consumption | 20117 | Self-reported alcohol drinker status (categorized as never, previous, and current |
| CHD | 6150 | Vascular or heart problems diagnosed by a physician (specifically self-reported heart attack or angina) |
| Stroke | 6150 | Vascular or heart problems diagnosed by a physician (specifically self-reported stroke) |
| Hypertension | 6150 | Vascular or heart problems diagnosed by a physician (specifically self-reported high blood pressure) |
| Diabetes | 2443 | Diabetes diagnosed by a physician |
| Depression | 41270 | Hospital inpatient diagnoses (ICD-10 codes: F32 [depressive episode] and F33 [recurrent depressive disorder]) |

**Table S2 Baseline Characteristics of the Study Populations of sub-SHAPE Cohort**

|  | SHAPE | | | | | |
| --- | --- | --- | --- | --- | --- | --- |
| **Characteristic** | **No condition**  **(n= 1,374)** | **Isolated insomnia**  **(n= 916)** | **Isolated anxiety**  **(n= 305)** | **Comorbid**  **(n= 460)** | **Total**  **(n= 3,055)** | **P Value** |
| **Age, mean (SD), y** | 71.2 (7.5) | 73.4 (8.0) | 73.2 (8.0) | 77.7 (9.4) | 73.0 (8.3) | <0.001 |
| **Female, No. (%)** | 622 (45.3) | 349 (38.1) | 141 (46.2) | 166 (36.1) | 1,278 (41.8) | <0.001 |
| **Education level, No. (%)** |  |  |  |  |  | <0.001 |
| **Low** | 473 (34.4) | 425 (46.4) | 121 (39.7) | 218 (47.4) | 1,237 (40.5) |  |
| **Intermediate** | 839 (61.1) | 433 (47.3) | 168 (55.1) | 222 (48.3) | 1,662 (54.4) |  |
| **High** | 62 (4.5) | 58 (6.3) | 16 (5.3) | 20 (4.3) | 156 (5.1) |  |
| **Current smoking, No. (%)** | 193 (14.1) | 115 (12.6) | 52 (17.1) | 74 (16.1) | 434 (14.2) | <0.001 |
| **Current alcohol consumption, No. (%)** | 128 (9.3) | 89 (9.7) | 33 (10.8) | 57 (12.4) | 307 (10.1) | 0.42 |
| **Coronary heart disease, No. (%)** | 59 (4.3) | 84 (9.2) | 29 (9.5) | 74 (16.1) | 246 (8.1) | <0.001 |
| **Stroke history, No. (%)** | 39 (2.8) | 50 (5.5) | 14 (4.6) | 37 (8.0) | 140 (4.6) | <0.001 |
| **Hypertension, No. (%)** | 381 (27.7) | 427 (46.6) | 115 (37.7) | 259 (56.3) | 1,182 (38.7) | <0.001 |
| **Diabetes, No. (%)** | 127 (9.2) | 123 (13.4) | 37 (12.1) | 144 (31.3) | 431 (14.1) | <0.001 |
| **Depression, No. (%)** | 90 (6.6) | 196 (21.4) | 127 (41.6) | 334 (72.6) | 747 (24.5) | <0.001 |
| **APOEε4 carrier, No. (%)** | 302 (22.0) | 222 (24.2) | 58 (19.0) | 116 (25.2) | 698 (22.9) | 0.23 |
| **TCSA score, mean (SD)** | 9.0 (2.3) | 8.0 (2.8) | 8.8 (2.3) | 7.0 (3.0) | 8.4 (2.7) | <0.001 |
| **Plasma P-tau217, median (IQR), pg/mL** | 1.62 (1.02, 2.55) | 1.71 (1.10, 2.69) | 1.66 (1.01, 2.65) | 2.46 (1.73, 3.89) | 1.76 (1.08, 2.79) | <0.001 |
| **Plasma NfL, median (IQR), pg/mL** | 14.09 (6.86, 22.16) | 13.46 (6.38, 21.59) | 14.32 (7.22, 22.90) | 15.36 (7.24, 24.42) | 14.27 (7.01, 22.62) | 0.27 |
| **Plasma GFAP, median (IQR), pg/mL** | 8.52 (5.48, 11.95) | 8.64 (5.48, 11.94) | 8.76 (5.66, 12.03) | 10.00 (6.63, 14.54) | 8.79 (5.67, 12.37) | <0.001 |
| **Cognitive impairment, No. (%)** | 275 (20.0) | 211 (23.0) | 92 (30.2) | 253 (55.0) | 831 (27.2) | <0.001 |

Data were presented as mean (SD), numbers and (percentages). Group comparisons for continuous variables were performed using the Kruskal-Wallis test. Categorical variables were compared using the Chi-square test (or Fisher's exact test when appropriate). Exposure phenotypes were strictly defined based on clinical status at baseline.

Abbreviations: UKB, UK Biobank; SD, standard deviation; TCSA, Thoven Cognitive Self-Assessment; CHD, coronary heart disease; APOE, apolipoprotein E.

**Table S3. Detailed interaction measures between comorbid insomnia and anxiety on incident cognitive impairment and dementia in the SHAPE and UK Biobank cohorts**

|  | **SHAPE Cohort** | |  | **UK Biobank Cohort** | | |
| --- | --- | --- | --- | --- | --- | --- |
| **Measures** | **Estimate** | **95% CI** | **P-value** | **Estimate** | **95% CI** | **P-value** |
| No Anxiety×No Insomnia | 1 | — | — | 1 | — | — |
| Anxiety×No Insomnia  No Anxiety×Insomnia | 0.90  1.00 | 0.73, 1.11  0.96, 1.04 | 0.326  0.864 | 1.09  0.95 | 0.80, 1.48  0.88, 1.02 | 0.576  0.123 |
| Anxiety×Insomnia | 2.07 | 1.84, 2.33 | <0.001 | 1.54 | 1.32, 1.79 | <0.001 |
| **Interaction Metrics** | | |  |  |  |  |
| Multiplicative interaction | 2.29 | 1.81, 2.90 | <0.001 | 1.49 | 1.07, 2.09 | 0.019 |
| Additive interaction | | |  |  |  |  |
| RERI | 1.17 | 0.87, 1.47 | <0.001 | 0.5 | 0.11, 0.90 | 0.006 |
| AP | 0.57 | 0.42, 0.71 | <0.001 | 0.33 | 0.09, 0.56 | 0.003 |
| SI | -11.22 | -34.40, 11.97 | / | 14.89 | 0.001, 1.95×10⁵ | / |

**Note: CI = Confidence Interval; RERI = Relative Excess Risk due to Interaction; AP = Attributable Proportion; SI = Synergy Index.**

**Table S4 Detailed Path Coefficients for the Parallel Mediation Models Across Groups**

| **Group** | **Path** | **Link** | **b (95% CI)** | **SE** | **P-value** |
| --- | --- | --- | --- | --- | --- |
| Isolated anxiety | Path a | Exposure to P-tau217 | 0.02 (-0.08 to 0.13) | 0.05 | 0.686 |
|  | Path a | Exposure to NfL | -0.12 (-0.41 to 0.18) | 0.15 | 0.436 |
|  | Path a | Exposure to GFAP | 0.08 (-0.07 to 0.22) | 0.07 | 0.304 |
|  | Path b | P-tau217 to Cognitive impairment | 0.78 (0.65 to 0.90) | 0.06 | <0.001 |
|  | Path b | NfL to Cognitive impairment | 0.03 (-0.01 to 0.07) | 0.02 | 0.178 |
|  | Path b | GFAP to Cognitive impairment | 0.13 (0.03 to 0.23) | 0.05 | 0.008 |
|  | Path c' | Direct Effect | 0.29 (-0.02 to 0.60) | 0.16 | 0.070 |
|  | Path c | Total Effect | 0.31 (0.00 to 0.63) | 0.16 | 0.050 |
| Isolated insomnia | Path a | Exposure to P-tau217 | -0.01 (-0.08 to 0.06) | 0.04 | 0.755 |
|  | Path a | Exposure to NfL | -0.02 (-0.22 to 0.18) | 0.10 | 0.831 |
|  | Path a | Exposure to GFAP | 0.00 (-0.10 to 0.10) | 0.05 | 0.945 |
|  | Path b | P-tau217 to Cognitive impairment | 0.78 (0.65 to 0.90) | 0.06 | <0.001 |
|  | Path b | NfL to Cognitive impairment | 0.03 (-0.01 to 0.07) | 0.02 | 0.178 |
|  | Path b | GFAP to Cognitive impairment | 0.13 (0.03 to 0.23) | 0.05 | 0.008 |
|  | Path c' | Direct Effect | -0.02 (-0.25 to 0.21) | 0.12 | 0.863 |
|  | Path c | Total Effect | -0.03 (-0.26 to 0.21) | 0.12 | 0.807 |
| Comorbidity | Path a | Exposure to P-tau217 | 0.42 (0.31 to 0.52) | 0.05 | <0.001 |
|  | Path a | Exposure to NfL | -0.14 (-0.43 to 0.16) | 0.15 | 0.356 |
|  | Path a | Exposure to GFAP | 0.24 (0.10 to 0.39) | 0.07 | 0.001 |
|  | Path b | P-tau217 to Cognitive impairment | 0.78 (0.65 to 0.90) | 0.06 | <0.001 |
|  | Path b | NfL to Cognitive impairment | 0.03 (-0.01 to 0.07) | 0.02 | 0.178 |
|  | Path b | GFAP to Cognitive impairment | 0.13 (0.03 to 0.23) | 0.05 | 0.008 |
|  | Path c' | Direct Effect | 0.75 (0.45 to 1.04) | 0.15 | <0.001 |
|  | Path c | Total Effect | 1.10 (0.78 to 1.41) | 0.16 | <0.001 |

Parallel multiple mediation models were estimated using a two-step regression framework. Path a coefficients (exposure → mediator) were derived from linear regression and are reported as unstandardized coefficients (b); path b (mediator → cognitive impairment), path c′ (direct effect), and path c (total effect) coefficients were derived from logistic regression and are reported on the log-odds scale. Each group (isolated anxiety, isolated insomnia, comorbidity) was modeled relative to the reference group (participants with neither anxiety nor insomnia). P-tau217, GFAP, and NfL were entered simultaneously as parallel mediators; path b coefficients are therefore mutually adjusted and identical across exposure groups within the same model. The total effect (path c) represents the overall association between each exposure and cognitive impairment, the direct effect (path c′) represents this association after accounting for all three mediators, and the indirect effect through each mediator was estimated as the product of the corresponding a and b coefficients (a × b). 95% confidence intervals were obtained using the bias-corrected and accelerated (BCa) bootstrap method with 5,000 resamples; intervals excluding zero were considered statistically significant. All models were adjusted for age, sex, education, smoking, BP, CHD ,alcohol, diabetes, stroke, depression and APOE ε4 status. Abbreviations: P-tau217, phosphorylated tau 217; GFAP, glial fibrillary acidic protein; NfL, neurofilament light chain; CI, confidence interval; SE, standard error.

**Table S5 The interaction effect of insomnia and anxiety on the cognitive impairment in SHAPE Cohort excluding participants with sleep medicine**

| **Variables** | **OR (95%CI)** | ***P*** |
| --- | --- | --- |
| **No Anxiety×No Insomnia**  **N=42,636** | 1 [Reference] | NA |
| **Anxiety× No Insomnia**  **N=446** | 0.90 (0.73-1.11) | 0.31 |
| **No Anxiety×Insomnia**  **N=16,822** | 0.99 (0.95-1.04) | 0.75 |
| **Anxiety×Insomnia**  **N=1,251** | 1.87 (1.64-2.13) | <0.001 |
| **Multiplicative scale** | 2.10 (1.64-2.67) | <0.001 |
| **RERI** | 0.98 (0.68-1.28) | <0.001 |
| **AP** | 0.52 (0.40-0.65) | <0.001 |

Adjusted for age, sex, education, smoking, drinking, diabetes, CHD, stroke, hypertension

Abbreviation: OR Odds Ratio, CI Confidence interval, CHD coronary heart disease

**Table S6 The interaction effect of insomnia and anxiety on the cognitive impairment in SHAPE Cohort excluding participants with stroke**

| **Variables** | **OR (95%CI)** | ***P*** |
| --- | --- | --- |
| **No Anxiety×No Insomnia**  **N=41,629** | 1 [Reference] | NA |
| **Anxiety× No Insomnia**  **N=422** | 0.89 (0.72-1.11) | 0.30 |
| **No Anxiety×Insomnia**  **N=19,485** | 1.00 (0.96-1.04) | 0.96 |
| **Anxiety×Insomnia**  **N=1,428** | 1.95 (1.72-2.20) | <0.001 |
| **Multiplicative scale** | 2.18 (1.71-2.78) | <0.001 |
| **RERI** | 1.05 (0.76-1.35) | <0.001 |
| **AP** | 0.54 (0.38-0.71) | <0.001 |

Adjusted for age, sex, education, smoking, drinking, diabetes, CHD, stroke, hypertension

Abbreviation: OR Odds Ratio, CI Confidence interval, CHD coronary heart disease

**Table S7 The interaction effect of insomnia and anxiety on the cognitive impairment in SHAPE Cohort excluding participants with low education**

| **Variables** | **OR (95%CI)** | ***P*** |
| --- | --- | --- |
| **No Anxiety×No Insomnia**  **N=38,075** | 1 [Reference] | NA |
| **Anxiety× No Insomnia**  **N=387** | 0.99 (0.80-1.24) | 0.96 |
| **No Anxiety×Insomnia**  **N=16,754** | 0.97 (0.93-1.01) | 0.21 |
| **Anxiety×Insomnia**  **N=1,297** | 1.93 (1.70-2.19) | <0.001 |
| **Multiplicative scale** | 1.99 (1.55-2.57) | <0.001 |
| **RERI** | 0.96 (0.64-1.28) | <0.001 |
| **AP** | 0.50 (0.32-0.68) | <0.001 |

Adjusted for age, sex, education, smoking, drinking, diabetes, CHD, stroke, hypertension

Abbreviation: OR Odds Ratio, CI Confidence interval, CHD coronary heart disease

**Table S8 The interaction effect of insomnia and anxiety on the dementia in UKB Cohort excluding incident cases within 5 years**

| **Variables** | **HR (95%CI)** | ***P*** |
| --- | --- | --- |
| **No Anxiety × No Insomnia**  **(n=103,689)** | 1 [Reference] | NA |
| **Anxiety× No Insomnia (n=821)** | 1.04 (0.75-1.45) | 0.79 |
| **No Anxiety×Insomnia**  **(n=41,836)** | 0.95 (0.88-1.02) | 0.15 |
| **Anxiety×Insomnia**  **(n=2,773)** | 1.62 (1.39-1.90) | <0.001 |
| **Multiplicative scale** | 1.64 (1.15-2.35) | 0.007 |
| **RERI** | 0.63 (0.22-1.04) | 0.001 |
| **AP** | 0.39 (0.16-0.62) | <0.001 |

Adjusted for age, sex, education, smoking, drinking, diabetes, CHD, stroke, hypertension, depression, *APOE*

Abbreviation: HR Hazard Ratio, CI Confidence interval, CHD coronary heart disease

**Table S9 The interaction effect of insomnia and anxiety on the dementia in UKB Cohort excluding participants with depression**

| **Variables** | **HR (95%CI)** | ***P*** |
| --- | --- | --- |
| **No Anxiety × No Insomnia**  **(n=100,256)** | 1 [Reference] | NA |
| **Anxiety× No Insomnia**  **(n=469)** | 0.92 (0.50-1.72) | 0.80 |
| **No Anxiety×Insomnia**  **(n=39,614)** | 0.98 (0.91-1.06) | 0.68 |
| **Anxiety×Insomnia**  **(n=1,576)** | 2.54 (2.05-3.13) | <0.001 |
| **Multiplicative scale** | 2.79 (1.45-5.39) | 0.002 |
| **RERI** | 1.63 (0.85-2.41) | <0.001 |
| **AP** | 0.64 (0.40-0.88) | <0.001 |

Adjusted for age, sex, education, smoking, drinking, diabetes, CHD, stroke, hypertension, *APOE*

Abbreviation: HR Hazard Ratio, CI Confidence interval, CHD coronary heart disease
