# Supplementary material for: Synergistic association of insomnia and anxiety with cognitive impairment and dementia in older adults
Source: Alzheimers Dement. 2026 Sep 23;22(9):e71853. doi: 10.1002/alz.71853 (PMC13598542; doi:10.1002/alz.71853)
Supplement: Supplementary file 4 [file ALZ-22-e71853-s004.docx]

**Supplementary material**

**Supplementary methods**

**Detailed Assessment in the SHAPE Cohort**

**Insomnia**

Insomnia was assessed using the 8-item Athens Insomnia Scale (AIS-8). Severity was categorized as follows: scores of less than 4 (no insomnia), 4 to 6 (suspected insomnia), and greater than 6 (clinically significant insomnia). For the primary analysis, scores of 6 or less were operationally defined as non-insomnia, whereas scores greater than 6 were defined as insomnia. Notably, participants reporting the current use of sleep-aid medication were automatically classified into the insomnia group, irrespective of their AIS-8 scores.

**Anxiety**

Anxiety symptoms were evaluated via the 7-item Generalized Anxiety Disorder scale (GAD-7), with established severity thresholds of 0 to 4 (no anxiety), 5 to 9 (mild), 10 to 14 (moderate), and 15 to 21 (severe). In this study, a cutoff score of greater than 4 was used to define the presence of clinically relevant anxiety. To further explore the impact of symptom severity, we also performed a semi-quantitative analysis. GAD-7 scores were also divided into three categories: no anxiety (0-4 points), mild anxiety (5-9 points), and moderate-to-severe anxiety (≥10 points).

**Depression**

Depressive symptoms were assessed using the 15-item Geriatric Depression Scale (GDS-15). A score of 5 or greater indicated the presence of clinically relevant depressive symptoms.

**Cognitive Function**

Cognitive function was evaluated using the Thoven Cognitive Self-Assessment (TCSA), a validated, self-developed electronic screening tool. The TCSA features a 2-stage diagnostic protocol. The initial rapid screening module (11 points; 1-3 minutes) applies education-stratified cutoffs to determine normal cognitive function: a score of 8 or greater for individuals with 12 or fewer years of education, and 9 or greater for those with more than 12 years of education. Due to the logistical constraints inherent in large-scale community screening, completion rates for the comprehensive 30-point secondary evaluation were limited. Therefore, to maximize data completeness and ensure consistency, the present study defined cognitive impairment based on the initial 11-point module in conjunction with final diagnostic determinations made by attending physicians.

**Definition of Covariates in the SHAPE Cohort**

In the SHAPE cohort, covariates included sociodemographic characteristics (age, sex, and education level), lifestyle factors (smoking status and alcohol consumption), and baseline clinical comorbidities. To perfectly align with the 3-tier education classification used in the UK Biobank, the original 5-level education system in the SHAPE cohort was harmonized as follows: "low" (less than primary school or primary school), "intermediate" (junior high school or senior high school), and "high" (college or above). Clinical covariates included self-reported histories of hypertension, diabetes, coronary heart disease, and stroke, which were ascertained during structured face-to-face clinical interviews.

**Detailed Laboratory Protocols**

**Single Molecule Immune Detection of Plasma Biomarkers**

Plasma concentrations of phosphorylated tau 217 (P-tau217), glial fibrillary acidic protein (GFAP), and neurofilament light (NfL) were measured using the AST-Sc-Lite automated platform (AstraBio, Suzhou, China). This system employs a magnetic bead–based sandwich fluorescence immunoassay using single-molecule imaging and detection (SMID) technology. Briefly, the SMID workflow proceeds as follows: (1) capture antibody-coated magnetic beads (1.2μm, Merck EM1-100/40) are mixed with plasma samples and incubated at 40°C for 6 minutes to capture target antigens; (2) detection antibodies labelled with nano-fluorophores are added and incubated at 40°C for 4 minutes to form sandwich immunocomplexes; (3) the mixture is transferred to a flow cell (2 mm × 2 mm × 60 mm), where a permanent magnet immobilizes the magnetic beads onto the bottom surface as a homogeneous, random single-layer array; (4) unbound fluorophores are washed away, and fluorescent images of the bead array are captured by an integrated camera with excitation light source; and (5) single-molecule fluorescent signals are enumerated for antigen quantitation against internal standard curves. The coefficient of variation for bead numbers across 10 repeated measurements was 1.2%, indicating stable bead array generation.

To further verify the platform's performance in our current study cohort, we conducted an additional dedicated head-to-head comparison of pTau217 quantification between the AstraBio platform and SIMOA: 95 randomly selected samples were tested in parallel on both platforms. Linear fitting of the paired measurement results yielded an R² of 0.8699, indicating a strong linear correlation between the two platforms in reflecting pTau217 level variations. The observed slope of ~7.73 is attributed to differences in the metrological traceability systems adopted by the two platforms, which is a systematic difference in absolute value quantification and does not affect the validity of relative association analysis within our study cohort.

Moreover, we evaluated the clinical diagnostic performance of pTau217 measured by the AstraBio platform in an independent clinical cohort including 40 Aβ-positive and 40 Aβ-negative participants (Aβ status confirmed by AV45 PET). As shown below, pTau217 concentrations were significantly elevated in the Aβ-positive group, with an area under the receiver operating characteristic curve (AUC) of 91.24% for distinguishing Aβ status, further supporting the excellent reliability and clinical application value of this platform.

**APOE Genotyping Protocol**

Genomic DNA isolation was performed on cryopreserved peripheral blood cell fractions. APOE allelic status (ε2, ε3, and ε4) was characterized by targeting single nucleotide polymorphisms rs429358 and rs7412 via a fluorescence-based quantitative polymerase chain reaction (qPCR) melt-curve assay. PCR amplification was carried out in a 10-μL reaction volume comprising 2.0 μL of template DNA (25 ng/μL), 1.0 μL of 1× PCR buffer, 0.8 μL of deoxynucleotide triphosphates (2 mM), 0.1 μL of Taq DNA polymerase (1 U), 1.2 μL of primer mix (0.5 μM), and 4.9 μL of double-distilled water. The thermal profile was executed on a SLAN-96S Real-Time PCR System (Shanghai Hongshi Medical Technology Co, Ltd) under the following conditions: an initial denaturation step at 95 °C for 5 minutes, followed by 50 cycles of 95 °C (20 seconds), 60 °C (30 seconds), and 72 °C (30 seconds; without fluorescence collection), and a final extension at 72 °C for 5 minutes. Genotyping was finalized through postamplification melting curve analysis, involving a 60-second denaturation at 95 °C, a 3-minute annealing phase at 45 °C, and a continuous fluorescence acquisition ramp from 45 °C to 70 °C.
